# Cross-Talk Signaling in Rice During Combined Drought and Bacterial Blight Stress

**DOI:** 10.3389/fpls.2019.00193

**Published:** 2019-03-06

**Authors:** Ramu S. Vemanna, Rahul Bakade, Pooja Bharti, M. K. Prasanna Kumar, Sheshshayee M. Sreeman, Muthappa Senthil-Kumar, Udayakumar Makarla

**Affiliations:** ^1^Department of Crop Physiology, University of Agriculture Sciences, Bengaluru, India; ^2^Regional Center for Biotechnology, Faridabad, India; ^3^Department of Plant Pathology, University of Agriculture Sciences, Bengaluru, India; ^4^National Institute of Plant Genome Research, New Delhi, India

**Keywords:** combined stress, rice, *Xanthomonas*, drought, QTL, protein synthesis

## Abstract

Due to climatic changes, rice crop is affected by moisture deficit stress and pathogens. Tissue water limitation besides reducing growth rates, also renders the crop susceptible to the infection by *Xanthomonas oryzae* pv. *oryzae* (*Xoo*) that causes bacterial leaf blight. Independently, both drought adaptation and *Xoo* resistance have been extensively studied. Though the cross-talk between drought and *Xoo* stress responses have been explored from individual stress studies, examining the combinatorial stress response is limited in rice. Recently published combined stress studies showed that under the combined stress, maintenance of carbon assimilation is hindered and such response is regulated by overlapping cellular mechanisms that are different from either of the individual stresses. Several receptors, MAP kinases, transcription factors, and ribosomal proteins, are predicted for playing a role in cellular homeostasis and protects cells from combined stress effects. Here we provide a critical analysis of these aspects using information from the recently published combined stress literature. This review is useful for researchers to comprehend combinatorial stress response of rice plants to drought and *Xoo*.

## Introduction

Plants are simultaneously exposed to diverse biotic and abiotic stresses that result in reduced yields in many crops (Atkinson et al., 2013; Narsai et al., 2013; Prasch and Sonnewald, 2013; Suzuki et al., 2014; Pandey et al., 2015b; Ramegowda and Senthil-kumar, 2015; Bahuguna et al., 2018). Rice is generally grown under puddled conditions, however, due to shortage of water availability, water saving technologies have been adapted for crop production (Lampayan et al., 2004). The unexpected drought has a significant impact on nearly 23 million hectares of rain-fed rice growing area in Southeast Asia. During these situations, many bacterial pathogens infect plants and further reduce the yield. A combined effect of bacterial blight (BB) caused by *Xanthomonas oryzae* pv. *oryzae* (*Xoo*) and drought situations in dry season cause significant yield losses in South Asia and South Africa (Ogawa, 1993; Verdier et al., 2012; Dixit et al., 2014). However, highest yield losses were reported in drought stress followed by temperature, weeds, and diseases (Pantuwan et al., 2000; Savary et al., 2012; Singh et al., 2012; Aghamolki et al., 2014; Ghadirnezhad and Fallah, 2014).

Water limitation with its effect on tissue water relations besides reducing growth rates also renders the crop susceptible to the infection by *Xanthomonas*. Both drought adaptation and *Xoo* resistance have been extensively studied with significant leads. Understanding the response of the crop to a combination of drought and BB is a relevant topic. The major premise emerged from the fact that some mechanisms leading to stress adaptation could have a common link through protecting plant metabolic efficiency under these stresses (Ramegowda and Senthil-kumar, 2015).

The combined simultaneous occurrences of abiotic and biotic stresses depend on the host resistance or susceptibility and also on the race of pathogens (Tippmann et al., 2006). The multiple stress occurrence and microclimate of plant-microbe interactions also influence the response of the host plant. Overlapping plant responses to drought and bacterial stress have been reported in Arabidopsis, rice, chickpea, and sunflower (Atkinson et al., 2013; Prasch and Sonnewald, 2013; Choudhary et al., 2016; Vemanna et al., 2016). There are several common changes in morphological, physiological traits and biochemical responses of plants to drought and pathogen stresses (Pandey et al., 2017). Leaf wilting, decrease in tiller number and biomass are common processes affected in both drought and bacterial infections in rice. However, increased root growth and reduced leaf expansion, stem elongation and leaf number are observed only under drought and localized lesions, patchy brown spots or pale yellow leaves were observed upon bacterial infection in rice. There are common and unique plant responses observed in response to both stresses when exposed independently. These symptoms could be common, which may serve as morphological observations to identify the combined stress response in rice. ABA and ethylene increases in plants with concomitant reduction of photosynthetic ability under combined stresses (Grimmer et al., 2012; Zhang and Sonnewald, 2017). In these conditions, antioxidant enzymes are accumulated to scavenge the ROS generated under stress. However, ROS accumulation under pathogen infection is the cause for a hypersensitive response suggesting that ROS play similar and opposite complex functions in plant adaptation under combined stresses. Sugars and polyamines are also accumulated for stress protection under combined stresses. All these mechanisms have relevance in imparting combined stress tolerance.

The stress tolerance mechanisms adapted by rice under combined stresses is diverse that include some common/shared and unique responses. The common visible effects include wilting, reduction in tiller number due to the blockage in xylem that reduces the water flow, which affects photosynthetic machinery (Fatima and Senthil-Kumar, 2017). Drought-induced low tissue water potential and lesions caused by bacterial infection further decreases the photosynthesis and reduce yield.

## Transcriptional Responses to Individual and Combined Stress Overlap

A comprehensive understanding of crosstalk or regulatory networks involved in unique or shared responses for either individual or multiple stresses is much-needed (Pandey et al., 2015a). A deluge in omics data has provided greater insight into the diverse aspects of spatiotemporal responses of stresses in plants. Only a limited amount of data is available in public domain for combined stresses, especially, for drought and *Xanthomonas* infection. The meta-analysis studies using transcriptome data from different plant species have identified shared genes which acts simultaneously or independently under different stress conditions (Shaik and Ramakrishna, 2013, 2014; Vemanna et al., 2016). Meta-analysis of eight different viruses infecting Arabidopsis revealed several regulatory genes which are competently connected to the plant defense response (Rodrigo et al., 2012). These meta-analysis data help in understanding the crosstalk of specific genes between stress conditions.

**Figure 1:**
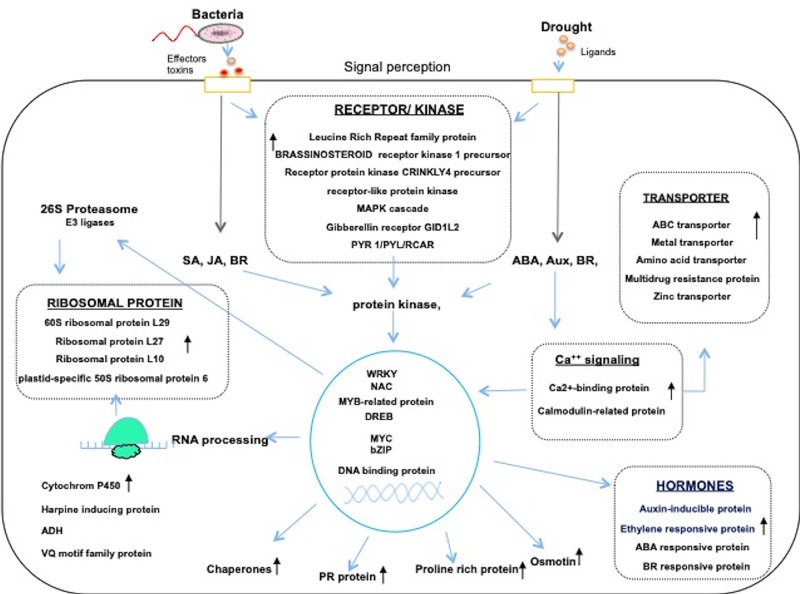
The molecular responses under drought and *Xoo* infection in Rice. The stress signals from biotic and abiotic stresses are perceived by specific receptors and cascade of signaling processes such as protien Kinases, TFs, transporters, ribosomal protiens, and many hormone responsive genes are differentially co-regulated in both drought and pathogen stress. The transcriptional regulators which play central role enhances the transcripts of diverse functional genes and ribosomal proteins translate the message in to protein to maintain cellular homeostasis under combined stress Many of these genes were upregulatcd under combined stress have relevance in improving stress adaptation (Narsai et al., 2013; Huang et al., 2014; Zhang et al., 2016).

Rice plants have evolved common molecular responses, which exhibit cross-talk between different hormones such as ABA, ethylene, salicylic acid, jasmonic acid, cytokinin, and brassinosteroid. The data from a few transcriptome analysis indicates the existence of crosstalk mechanisms between signaling networks under drought and pathogen stress (Figure 1) (Schenk et al., 2000; Cheong et al., 2002; Seki et al., 2002). Hormones play a crucial role as central regulators of many downstream responsive transcription factors (TFs) and functional proteins. The receptors for abscisic acid (ABA), brassinosteroids (BRs) and many pathogens triggered elicitors have been identified. Some are *PYR1/PYL/RCAR, BAK1*, and *LRR kinases* which act as *R* genes for many pathogens and also acts as key receptors in abiotic stress signaling (Figure 1). The signals received by these elicitors activates or phosphorylate the downstream protein kinases cascade to activate several TFs. The members of WRKY, NAC, AP2/ERF, bZIP, and MYC family TFs showed altered responses to both biotic and abiotic stresses (Babitha et al., 2013, 2015a,b; Xiao et al., 2013; Zhang et al., 2016; Ku et al., 2018) and played a major role in combined stresses. The reactive oxygen species (ROS) generated under oxidative stress showed unique responses to bacterial and drought stresses that trigger downstream stress responses (Apel and Hirt, 2004; Narusaka et al., 2004; Torres and Dangl, 2005). The calcium signaling is considered as a central hub in concurrent biotic and abiotic stress responses (Ranty et al., 2016; Ku et al., 2018). The signals of Ca^2+^, inositol-3-phosphate and protein kinases and other kinases also have a significant role in combined stresses. Thus crosstalk between biotic and abiotic stress signaling pathways regulate many cellular processes.

The individual transcriptome data from biotic and abiotic stress have identified antagonistic and overlapping responses (Narsai et al., 2013). The computational comparison of the expression profile between abiotic and biotic stresses has revealed unique genes that showed similar response across multiple stresses (Jain et al., 2007; Ribot et al., 2008; Swarbrick et al., 2008; Hu et al., 2009; Marcel et al., 2010; Zhou et al., 2010; Narsai et al., 2013). The studies suggest that the plants respond to combined multiple stresses by crosstalk of several hormonal signaling pathways (Sharma et al., 2013; Bahuguna et al., 2018; Ku et al., 2018). In response to drought and *Xoo* infection, 2276 genes showed overlapping differential expression profiles (Narsai et al., 2013). In another, meta-analysis study between drought and bacterial stress in rice 5084 DEGs have been identified by combining the data sets. Among 1214 common genes, 565 genes were upregulated and 309 downregulated in both drought and bacterial stresses (Shaik and Ramakrishna, 2013). In a comparative study of drought and *Xoo*, transcriptome analysis of the resistant rice introgressed line H471 when compared with the recurrent parent HHZ and 306 and 840 DEGs were identified and amongst them 178 genes were common for both stresses (Zhang et al., 2016).

In combined drought and *Xoo* infection, many components of the multiple pathways responded similarly. Several genes showed opposite roles in response to pathogens and abiotic stress tolerance (Xiong and Yang, 2003; Asano et al., 2012). The *OsCPK12* acts as a negative regulator for blast resistance but positively regulates salt, drought, and cold stress tolerance in rice (Asano et al., 2012). The broad-spectrum disease resistance through *PR* genes is negatively regulated by *OsMAPK5* (Xiong and Yang, 2003). In resistant introgressed lines H471, two genes (*LOC_Os04g56000* and *LOC_Os12g43410*), were strikingly up and down-regulated in combined drought and *Xoo* infection. The studies suggest that the drought tolerance and BB resistance mechanisms are shared in resistance genotypes. Further, phosphate/phosphorus metabolic process, phosphotransferase activity, and kinase activity associated genes and peptidase/endopeptidase/enzyme inhibitor activity genes were highly represented in up-regulated genes. The TFs *WRKY* and *NAC* showed a conserved response between abiotic and biotic stress (Seki et al., 2002; Narsai et al., 2013). Under both stress conditions, 10 DEGs encoding receptor kinases *LOC_Os08g07760 (OsBAK1)*, protein kinases *LOC_Os11g31530* (brassinosteroid signaling pathway) were strongly up-regulated and *LOC_Os03g46910* (pyruvate kinase) was down-regulated in H471 introgression line which is resistant to *Xoo* and drought as compared with Huang-Hua-Zhan (HHZ) (Zhang et al., 2016).

In rice, the introgressed line H471, three DEGs involved in phytohormone signaling pathways, the BR pathway (*OsBAK1*) and gibberellin (GA) pathway genes were upregulated in both drought and *Xoo* stress. The *GA20 oxidase* an essential gene involved in GA biosynthesis that catalyzes the conversion of GA53 to GA20 was downregulated (Zhang et al., 2016). The GA response related to plant height was evident, and the response could be associated with *GA20 oxidase* expression levels (Dossa et al., 2016) The antagonistic reaction from GA with JA has been reported that they are involved in development and immunity of plants through DELLA proteins (Yang et al., 2013).

The stress-responsive signaling genes are differentially regulated in response to combined stresses. The ABC transporters such as multidrug resistance function encoding proteins, universal stress protein (*LOC_Os5g28740*), Q-rich domain-containing protein (*LOC_Os06g04240*) and lineage-specific genes (*LOC*_*Os12g32610*) were highly expressed in both stresses in rice signifying the importance of these transporters in combined stresses. In *Xoo* resistant genotypes, cell wall-associated genes were downregulated in 24 h of infection and significantly upregulated in 96 h. The phenylpropanoid metabolism genes UDP glucosyl/glucoronyl transferases, two genes encoding cytochrome 450 72A1 were significantly up-regulated in *Xoo* resistant type. There are receptor kinases such as *OsWAK (OsWAK127)*, a lectin-like receptor kinase, a phytosulfokine receptor precursor and a serine/ threonine kinase-like protein, an NBS-LRR type putative disease resistance protein (*LOC_Os02g30150*) and resistance protein *LR10* (*LOC_Os04g11780*) were up-regulated in response to *Xoo* infection, several of kinases were down-regulated in drought stress suggesting that there are specific receptor kinases exclusively responsive to individual stresses suggesting the unique signaling pathways may operate for stress adaptation by regulating downstream TFs.

The functional roles of some TFs have been elucidated in response to bacterial infection and drought stress. The TFs such as *WRKY28, MYB4, AP2/EREBP- DREB*, and *HSF4* were differentially regulated that control several functional genes involved in multiple stresses. The *C3H12 zinc finger* TF in downregulated at 96 h in response to bacterial *Xoo* infection (Narsai et al., 2013) and knock-out lines showed partially increased susceptibility in Zhonghu 11 genotype (Deng et al., 2012). Three *WRKY* TFs were up-regulated and had been shown to result in altered resistance. Overexpression of *WRKY71* resulted in enhanced resistance to *Xoo* bacterial infection (Liu et al., 2007). In contrast, over-expression of *WRKY45* showed increased susceptibility to *Xoo* (Tao et al., 2009). The *NAC TFs* that were upregulated in both abiotic and biotic stress also had a developmental role in plants (Hu et al., 2006; Mao et al., 2007; Tao et al., 2009; Jeong et al., 2010; Takasaki et al., 2010). Some *NAC TFs* were induced in response to *Xoo* infection and drought stress, amongst them *NAC10* showed 53-fold induction in drought stress (Jain et al., 2007) and overexpression resulted in root enlargement and improved drought stress tolerance (Jeong et al., 2010). The *bHLH* (LOC_Os01g72370), *B3* (LOC_Os03g42280), and M-type (LOC_Os04g31804) TFs were up-regulated and CO-like (LOC_Os09g06464) TF was down-regulated under drought and *Xoo* infections in resistant H471 rice genotype (Zhang et al., 2016). The *VQ* genes (VQ -FxxxVQxLTG motif) were shown to interact with WRKY TFs and were induced upon *Xoo* infection, ABA and drought stress conditions (Kim et al., 2013). The differential expression of TFs may regulate diverse functional genes, which have specific mechanisms under both stress conditions.

The cytochrome P450 monooxygenase family *CYP71P1* encoding tryptamine 5-hydroxylase function involved in cell wall biosynthesis was highly upregulated in response to *Xoo* infection as well as fungus *Magnaporthe oryzae* causing Sekiguchi lesion (SL) (Fujiwara et al., 2010; Delteil et al., 2012). The *SPL7* and *BiP3* chaperons were up-regulated in resistant genotypes in response to *Xoo* infection and drought. Suppression of SPL7 resulted in increased resistance to infection (Yamanouchi et al., 2002) and *Xa21* mediated immunity to *Xoo* infection was compromised due to overexpression of *BiP3* (Park et al., 2010). Binding proteins (BiP) play chaperone functions in endoplasmic reticulum-mediated unfolded protein response, improves cellular tolerance mechanisms by maintaining the protein quality control. The genes encoding cell cycle isomerases were differentially expressed (Yao et al., 2001) in the resistant genotype when infected with bacteria and among them, eight of them were highly upregulated in response to bacteria (Narsai et al., 2013). The pathogenesis-related *PR10* was induced in roots upon drought, salt stress, JA and blast fungus (Hashimoto et al., 2004).

The genes involved in protein degradation showed differential expression in combined stresses. The ubiquitin E3 complex and subtilizes were down-regulated in the resistant genotype in response to bacterial infection and drought. The speckle-type POZ protein (LOC_Os10g29220.1) a subunit of E3 ubiquitin complex and a subtilisin-like protease precursor (LOC_Os04g02970.1), deaminase (LOC_Os07g46630.1) involved in nucleotide degradation were downregulated in a bacterial infection in the drought-resistant cultivar. These E3 ligases are components of the 26S proteasome system are targeted by bacterial effector proteins and modulate their mechanisms against host defenses. The reduced expression of these genes in drought-resistant genotypes could be an adaptive strategy that plants have evolved to fight against *Xoo* infection.

## Regulatory Networks Under Combined Stress

**Figure 2:**
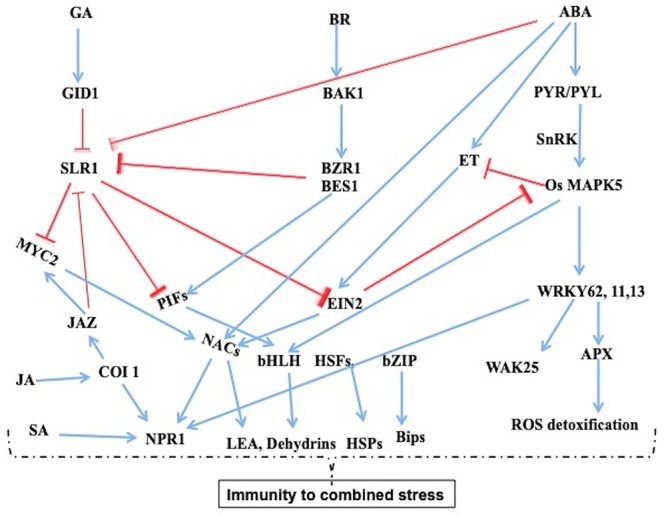
Regulatory networks under combined stress of drought and *Xoo* infection in nee. The data taken from the combined stress transcriptomic studies and a model arrived from the knowledge from diverse studies. The phytohormones play crucial role in crosstalk signaling mechanisms. Some of the key genes play antogonistic, overlapping, and opposite role with response to combined stress which depends on intensity of stress and cellular homeostasis with regards protein turnover or stability. The LEAs. dchydrins, HSPs, Bips provide stability to proteins (Narsai et al., 2013; Sharma et al., 2013).

Considerable crosstalk signaling mechanisms exist in response to combined bacterial and drought stress (Figure 2). In comparison with resistant rice introgressed line H471 and its recurrent parent HHZ, 178 common DEGs exists, among which 39 genes were found to be co-regulated in a complex network. Majority of proteins enriched belonged to stress signal perception and transduction such as *RLK, LRR, receptor kinases, protein kinases*, and the proteins were related to *BR (OsBAK1)* and *GA* (*GID1L2*) pathways (Zhang et al., 2016). There is convincing evidence to show that certain *RLKs* and *LRR*s are predicted to be involved in BR signaling process, which suggests that brassinosteroids act as a central regulatory hormone in crosstalk mechanisms with other hormone signaling process under combined studies. Several protein kinases involved in phosphorylation have been identified in both BB and drought tolerance, which further activates the TFs. However, several genes showed opposite roles in response to different stresses in rice (Xiong and Yang, 2003; Tao et al., 2009; Asano et al., 2012). The *Mitogen-Activated Protein Kinase 5 (OsMPK5)*, *wall-associated kinase 25 (WAK25), WAK-like (WAKL)*, *sucrose non-fermenting-1-related protein kinase-1 (SnRK1)* and *SUB1A* binding protein 23 (*SAB23*) are involved in cross-talk signaling in both abiotic and biotic interactions (He et al., 1998; Kohorn and Kohorn, 2012; Sharma et al., 2013). Suppression of *OsMPK5* reduced the ABA sensitivity and increased ethylene levels, PR protein expression, hence resulted in resistance to fungus *M. oryzae*, which causes rice blast disease (Xiong and Yang, 2003; Bailey et al., 2009). *SnRK1* has been identified as a central hub for signal integration for many pathways in the cross-talk mechanisms (Seo et al., 2011; Cho et al., 2012).

Downstream to these kinases several TFs are found to be commonly upregulated in both bacterial and drought stress which include HLH-type TF identified from combined stress (Zhang et al., 2016). Several TFs like *NAC, WRKY, MYB, MYC, bZIP, HSFs*, and *CO* identified were known to be upregulated in both stresses and induce immune responsive genes. Overexpression of *OsWRKY13* regulated SA-dependent immunity and several other physiological pathways including JA response. The *SNAC1* TF involved in abiotic stress response showed improved tolerance in rice (Qiu et al., 2007). Similarly other WRKY family TFs *OsWRKY45-1, OsWRKY62*, *71*, and *76* have an interface of the biotic and abiotic stress interactomes (Qiu and Yu, 2009; Seo et al., 2011). A few ribosomal protein-encoding genes, which include metal ion transport-related genes, PR protein osmotin, and GA associated genes, were co-regulated in resistant rice H471 type (Zhang et al., 2016). Several genes involved in photosynthesis, dehydrins and late embryogenesis abundant (LEA) proteins involved in protein stability that are implicated in desiccation tolerance are also co-regulated in bacterial stress (Hand et al., 2011; Shaik and Ramakrishna, 2013). ABA contributes to adaptation to osmotic stress and also involved in defense response by regulating plant physiological process which acts as a barrier for pathogen entry (Asselbergh et al., 2008; Ton et al., 2009; Kaundal et al., 2017). The *BAK1 and DELLA* proteins appears to be central regulators in abiotic and biotic stresses that positively affects ROS detoxification by activating many antioxidant proteins (Achard et al., 2008; Divi et al., 2010; Albrecht et al., 2012; Belkhadir et al., 2012; Sharma et al., 2013). In Arabidopsis, it was shown that the BR receptor *BAK1* is a primary signaling receptor that modulates the interaction of GA, BR, JA, and SA signaling pathways (Figure 2) (Yamada et al., 2015; Li et al., 2016; Nolan et al., 2017). DELLAs also sensitize JA signaling at the expense of SA mediated defense and enhances resistance to necrotrophic pathogens (Navarro et al., 2008). These studies demonstrate that the complex overlapping co-regulatory network of many pathways and genes contribute for adaptation to the combined stress conditions (Figure 2).

## Ribosomal Protein-Encoding Genes Are Differentially Regulated Under Combined Stress

Many omics reports over-representing genes associated with translational mechanisms such as TFs, RNA processing, RNA binding, ribosomal proteins, protein synthesis, and folding were differentially expressed. Recent studies show that the ribosomal protein-encoding genes have extra-ribosomal functions and they are involved in specific mechanisms (Nagaraj et al., 2016). Ribosomal proteins are critical for the translation of diverse proteins and involved in the overall fitness of the cell under stress conditions. From this context response of genes encoding ribosomal proteins were specifically looked in the combined stress conditions. However, the functional relevance of many of these genes with response to either individual or combined stresses is still needed. In response to bacterial infection, 50 genes encoding ribosomal proteins were up-regulated in the resistant rice genotype, and 46 of them were also up-regulated under fungal infection (*M. grisea*). However, in drought stress, 46 ribosomal proteins were downregulated (Narsai et al., 2013). The differential responses of all the ribosomal proteins including small and large subunit encoding proteins have been studied using genome-wide studies in rice with response to multiple stress condition (Moin et al., 2016; Saha et al., 2017). The ribosomal large subunit protein encoding genes were differentially expressed in response to abiotic stresses, and amongst them, 34 genes showed significant changes. Out of which, 6 of them were *RPL12, 28, 30*, 36, 44, and 51 that showed down-regulation in response to *Xoo*. *RPL38* was unchanged, and the remaining genes like *RPL10, 11, 15, 24a, 26, 27, 37* were activated more than 10-fold (Moin et al., 2016). The qRT-PCR analysis of small subunit encoding ribosomal protein genes revealed 14 genes downregulated, and others were upregulated in response to biotic and abiotic stresses. The *RPS6a, RPS9, RPS10a*, and *RPS4* showed high upregulation in both biotic and abiotic stresses (Saha et al., 2017). It was observed that out of 50, 32 ribosomal protein promoters have TELOBOX elements (Narsai et al., 2013). The differential regulation of these genes suggests that, unlike there role in protein synthesis, they also possess extra-ribosomal functions. The precise function of each ribosomal proteins with response to combined or individual stresses need to be identified.

## Introgression of Qtls for Drought and *Xa* Genes Improves Combined Stress Tolerance

Considerable progress has been made in identifying QTLs for drought tolerance (Prince et al., 2015). Similarly, around fifty genes /QTLs for resistance against *Xanthomonas* bacteria have been identified in rice (Dossa et al., 2016). Most of the genes are targeted to *Xoo* that causes BB and are referred to as *Xa* genes (Khan et al., 2014; Zhang et al., 2014). The *Xa21* being a major gene conferred resistance against bacterial infections, and subsequently, introgression of different *Xa* genes (*Xa5, Xa13, and Xa21*) provided broad-spectrum resistance in different rice cultivars (Pradhan et al., 2015). Drought stress influences plant response to pathogens through a gene for gene interaction and depends on the severity of stress. The combined interactive effect of bacterial disease and drought QTLs are dependent on QTLs or genes associated with specific traits. However, the combined stress response depends on soil water content and genotypes having different *Xa* genes (Wright and Beattie, 2004; Dossa et al., 2016). To date, no QTLs have been identified for combined bacterial and drought stress tolerance in rice. A few studies have shown genotype dependent BB pathogen infection in rice plants under drought-induced conditions (Dossa et al., 2016). Though many genotypes have different *Xa* genes, increased lesions were visible under mild drought stress conditions, indicating under combined stress, rice plants are affected and bacterial virulence enhanced.

The combined stress effect at vegetative stage showed different lesion length upon BB infection under drought stress which depends on genotypes having different *Xa* genes. The genotype containing single *Xa*4 gene and drought QTL -DTY2.2 did not show any significant BB induced lesion in either control or at severe drought conditions (Dossa et al., 2016). The susceptible IR24, IR64, and other two *Xa* gene introgressed lines had reduced lesion lengths under moderate drought stress. The multiplication and spread of *Xoo* were increased in rice genotypes under mild drought stress even though *Xa4* gene was expressed. However, under both compatible and incompatible interactions, BB disease infection was reduced under drought stress when drought severity was increased. The lesions in the single *Xa4* gene containing genotypes were larger than the genotypes having *Xa*7 gene when inoculated with virulent PXO145 (avr*Xa4* + avr*Xa5* + avr*Xa7*) indicating that the genotypes with suitable *Xa* gene may still provide resistance against the pathogen under drought stress conditions. Rice genotypes having different *Xa7, Xa4 + Xa7, Xa4 + Xa5 + Xa7*, and *Xa4*/ qDTY2.2 showed less disease development under drought stress. However, the genotypes having *Xa4, Xa4*/ qDTY2.2 + qDTY4.1 were less effective to combined stresses. Severe drought stress reduces the bacterial multiplication due to higher leaf water loss. The rice genotype with *Xa7* showed reduced bacterial multiplication under severe drought stress and was dependent on the tissue water status. Under severe drought stress, the leaf water loss is more which influences bacterial multiplication (Freeman and Beattie, 2009).

Single *Xa* gene is not sufficient to provide resistance. The rice genotype carrying single *Xa4* gene showed increased BB severity under drought stress. The compromised resistance response was also observed in rice genotype having *Xa4* gene under high temperature (Webb et al., 2010) and drought stress (Dossa et al., 2016). Similar reports were found in the combined stress of high temperature and BB, drought stress and BB at the seedling stage. From this context, the QTLs associated with drought tolerance with multiple *Xa* genes at the seedling level may improve tolerance for combined stress. From this context, introgression of *Xa* genes with drought QTLs signifies that the drought-tolerant genotypes with specific QTLs could be beneficial for BB disease development. The drought tolerant genotypes that can maintain water loss by regulating stomata or by deep roots with specific *Xa* genes could contribute to BB tolerance and improve combined stress tolerance. Higher stomata and root hydraulic conductivity under drought showed inhibition of BB in rice genotypes having three different *Xa* genes (Yu et al., 2008, 2013; Henry et al., 2015). Studies demonstrated that, when two major *R* genes (*Xa4 and Xa7*) are present in a genotype, combined stress tolerance is enhanced. In a recent study to improve the multiple stress tolerance, four BB resistance genes (*Xa 4*, *xa5*, *xa13*, *Xa21*) were pyramided with submergence (*Sub1*), salinity (*Saltol*), blast (*Pi2*, *Pi9*) and gall midge (*Gm1*, *Gm4*) improved Tapaswini an elite rice cultivar successfully that showed multiple stress tolerance (Das et al., 2018). The enhanced rice resistance to combined stress can be achieved by introgression of multiple drought QTLs and multiple R genes in a single elite genotype.

## Genetic Manipulation for Combined Stress Tolerance

Several genes have been identified and functionally characterized for their role in specific pathways and stress responses. However, most of these studies were limited to the single type of stresses and this could be considered as a major limitation in transgenic research aimed for product development, as the plants were not evaluated under combined stress conditions that occur in the natural environmental conditions. Here, we list a few genes that are tested for multiple individual stresses that showed differential roles (Supplementary Table 1). However, none of these genes were tested for combined stress response, and hence the data presented here is only a speculation that these genes may provide tolerance.

Overexpression of *MoHrip1 and MoHrip2* from *M. oryzae* in rice enhanced the resistance to bacterial disease caused by *Xoo* and drought stress. In transgenic plants, higher expression of two JA/ethylene biosynthesis-related genes *OsLOX2* and *OsAOS2* and SA signal-related genes *OsEDS1, OsPAL1, OsNH1, OsPR-1a*, and *OsPR-10a* was observed in response to bacterial pathogen and abiotic stress responsive genes *OsbZIP23, OsZEP1, OsNCED2*, and *OsNCED3* were highly upregulated under drought conditions (Wang et al., 2017). A few TFs from *NAC*, *WRKY*, *bHLH*, *AP2*, and *bZIP* family have been shown to be induced upon both drought and bacterial stresses (Nakashima et al., 2007; Xiao et al., 2013; Jisha et al., 2015). The *WRKY45-2* TF showed broad-spectrum disease resistance to *M. oryzae*, bacterial pathogens *Xoo* and *Xanthomonas oryzae* pv. *oryzicola.* However, this TF had been shown to act as a negative regulator of salt, cold, and drought stresses in rice (Tao et al., 2009, 2011). Similarly, overexpression of *WRKY13* enhanced rice resistance to *Xoo* and *M. oryzae* and reduced resistance to cold and salt stresses by influencing the transcription of more than 500 genes (Qiu et al., 2007, 2008). Transcriptional repressor *WRKY13* suppresses the expression of two important genes *SNAC1* and *WRKY45-1* by binding to sequence-specific W-like-type *cis*-elements on the promoters of these genes under abiotic and biotic stress. The autoregulation of *WRKY13* is associated with balancing its function when the rice plants experience different stress environments (Xiao et al., 2013). Ectopic expression of *OsWRKY11* resulted in up-regulation of defense-associated genes and drought-responsive genes that improve stress tolerance. *OsWRKY11* play positive regulator function in plant defense to drought and *Xoo* (Lee et al., 2018). Overexpression and suppression of a few specific genes resulted in resistance to combined biotic and abiotic stresses (Zhang et al., 2016). These studies demonstrate that the transcriptional regulators play a crucial role in improving multiple stress tolerance in rice. However, their response to combined stress needs to be assessed to gain more insight into their role in enhancing adaptation to natural environmental stresses. The genes that showed tolerance to both drought and *Xoo* could be attractive targets for genetic manipulation of rice for combined stress.

## Conclusion and Perspective

1. The plant responses under combined drought and bacterial infection need further understanding and studies using simultaneous stress imposition are much needed.
2. The existing transcriptome studies suggest combined stress responses are complex and sophisticated and hence detailed understanding of unique and shared signaling mechanisms is important.
3. Prospecting the candidate genes and functional validation using diverse approaches may lead to developing durable, resistant rice for combined bacterial and drought stress.
4. Understanding the regulatory networks involved in combined stress responses may provide an option to manipulate the signaling mechanisms which serve as a key for adaptation by using novel approaches such as genome editing tools.
5. Ribosomal protein-encoding genes seem to be attractive candidates for gene manipulation. However, the functional relevance in combined stress needs to be explored.
6. Combining drought QTLs and *Xa* genes could be a better strategy due to their success in the drought-prone areas. However, there is a need to identify QTLs at different stages of crop growth and develop introgressed lines, which may provide an option to improve rice for combined stress tolerance.
7. Pyramiding multiple genes/QTLs associated with multiple stresses in the elite background may provide durable resistance to combined stress.
8. Transgenics using candidate genes, which provide combined stress tolerance, are the best option because of their precise molecular mechanisms. However, more concerted efforts are needed to explore the candidate genes.
9. The alternate strategies like discovery of novel small molecules or dsRNA-mediated approaches can be employed to improve combined stress tolerance.

## Author Contributions

RV conceived the concept and wrote a review. RV, RB, and PB drafted the manuscript. MK edited the pathogen-related and SS edited the drought-related aspects in manuscript. MS-K, UM, and RV edited and finalized the manuscript.

## Conflict of Interest Statement

The authors declare that the research was conducted in the absence of any commercial or financial relationships that could be construed as a potential conflict of interest.
