# Supplementary material for: Cross-Talk Signaling in Rice During Combined Drought and Bacterial Blight Stress
Source: Front Plant Sci. 2019 Mar 6;10:193. doi: 10.3389/fpls.2019.00193 (PMC6415615; doi:10.3389/fpls.2019.00193)
Supplement: Supplementary file 1 [file Table_1.docx]

**Suplementary Table 1.** List of candidate genes that have relevance in improving combined stress tolerance of bacterial blight and drought stress in rice.

| Sl. No. | Gene name | Function | Expression  OX (over) / Down/ | Remark | Reference |
| --- | --- | --- | --- | --- | --- |
| 1 | *LOX 3*  *LOC_Os03g49350* | lipoxygenase | Antisense construct | Resulted in Embryo Lox3 deletion and reduced expression and sensitive to rice blast, bacterial blight and drought stress | Liu N. N. et al., 2008 |
| 2 | *Os2H16* | Induced upon Xoo, fungal and drought stresses | OX/RNAi | Transgenic lines showed enhanced tolerance to bacterial blight and sheath blight diseases and drought stress | Li N. 2013 |
| 3 | *OsMADS26* | Transcription factor activity, involved in developmental program | OX/ RNAi | Down-regulated plants exhibit enhanced resistance against  *Magnaporthe oryzae* and *Xanthomonas oryzae* and showed tolerance to water deficit. | Khong N.K. 2015 |
| 4 | *OsEREBP1 (AP2/ERF Type TF*) | TF, localized plastid nucleoids | OX | In transgenic plants upregulation of *lipase, chl lipoxigenase, JA, ABA* biosynthesis, *PR, ALDHs* genes observed that resulted in enhanced tolerance to drought and Xoo | Jisha et al., 2015 |
| 5 | *MPKK10.2* | Mitogen-activated protein kinase | OX/RNAi | Phosphorylate and activate MPK6 and MPK3. Overexpression plants showed tolerance to Xoc and drought and RNAi plants are sensitive | Ma et al., 2017 |
| 6 | *WRKY13* | Autoregulated TF acts as repressor by binding to SNAC1 and WRKY45-1 genes promoters. | OX/RNAi | Overexpression plants are sensitive to drought and Xoo. RNAi plants are tolerant because SNAC1 regulate stomata and WRKY45-1 shows enhanced tolerance to bacterial diseases. | Xiao et al., 2013 |
| 7 | *OsHsp18.0* | Class II small heat shock protein involved in nucleo-cytoplasmic trafficking | OX/ RNAi | Enhanced Xoo resistance , heat and salt treatments by overexpression of *OsHsp18.0* through  salicylic acid-dependent defense responses | Kuang et al., 2017 |
| 8 | *OsNAC6* | NAC TF family | OX | Transgenic rice plants showed an improved tolerance to dehydration and high-salt stresses, and also exhibited increased tolerance to blast disease. | Nakashima et al., 2007 |
| 9 | *OsHAP2E* | Rice heme activator protein gene-known to be a nuclear factor Y or CCAAT binding factor (HAP/NFY/  CBF), | OX | Regulate plant growth, development and stress responses. Confers resistance to pathogens, salinity and  drought, increases photosynthesis and tiller number | Alam et al.. 2015 |
| 10 | *OsAPX8* | Rice thylakoid membrane bound  ascorbate peroxidase | OX/RNAi | *OsAPX*8 responds to both bacterial and abiotic stresses by activating *WRKY* and cell wall tightening to trigger basal defense by scavenge excess H202 in chloroplast. | Jiang et al., 2016 |
| 11 | *OsMAPK5* | Mitogen-activated protein kinase | OX/RNAi | Suppression of *OsMAPK5* reduces kinase activity and enhanced expression of pathogenesis-related *PR1* and *PR10* genes resulted in resistance to fungal (*Magnaporthe grisea*) and bacterial (*Burkholderia glumae)* pathogens. Where as Overexpressed plants showed enhanced drought, salt, and cold tolerance. | Xiong and Yang et al., 2003. |
| 12 | *MoHrip1* and *MoHrip2* | Isolated from pathogenic fungus *Magnaporthe oryzae (M. oryzae*), protein elicitors | OX | Transgenic rice plants displayed higher resistance to rice blast  and tolerance to drought stress | Wang et al., 2017 |
| 13 | WARKY 11 | Binding to promoters of  both biotic and abiotic stress-related genes | OX/RNAi | Overexpressed lines showed enhanced resistance to Xoo by upregulating defense related genes and the knockdown lines showed compromised resistance | Lee et al., 2018 |

References

# [Alam, M.M](https://www.ncbi.nlm.nih.gov/pubmed/?term=Alam%20MM%5BAuthor%5D&cauthor=true&cauthor_uid=25168932)., [Tanaka, T](https://www.ncbi.nlm.nih.gov/pubmed/?term=Tanaka%20T%5BAuthor%5D&cauthor=true&cauthor_uid=25168932)., [Nakamura, H](https://www.ncbi.nlm.nih.gov/pubmed/?term=Nakamura%20H%5BAuthor%5D&cauthor=true&cauthor_uid=25168932)., [Ichikawa, H](https://www.ncbi.nlm.nih.gov/pubmed/?term=Ichikawa%20H%5BAuthor%5D&cauthor=true&cauthor_uid=25168932)., [Kobayashi, K](https://www.ncbi.nlm.nih.gov/pubmed/?term=Kobayashi%20K%5BAuthor%5D&cauthor=true&cauthor_uid=25168932)., [Yaeno, T](https://www.ncbi.nlm.nih.gov/pubmed/?term=Yaeno%20T%5BAuthor%5D&cauthor=true&cauthor_uid=25168932)., et al. (2015). Overexpression of a rice heme activator protein gene (OsHAP2E) confers resistance to pathogens, salinity and drought, and increases photosynthesis and tiller number. *[Plant Biotechnol J.](https://www.ncbi.nlm.nih.gov/pubmed/25168932)* 13, 85-96. doi: 10.1111/pbi.12239.

# [Jiang, G](https://www.ncbi.nlm.nih.gov/pubmed/?term=Jiang%20G%5BAuthor%5D&cauthor=true&cauthor_uid=27185545)., [Yin, D](https://www.ncbi.nlm.nih.gov/pubmed/?term=Yin%20D%5BAuthor%5D&cauthor=true&cauthor_uid=27185545)., [Zhao, J](https://www.ncbi.nlm.nih.gov/pubmed/?term=Zhao%20J%5BAuthor%5D&cauthor=true&cauthor_uid=27185545)., [Chen, H](https://www.ncbi.nlm.nih.gov/pubmed/?term=Chen%20H%5BAuthor%5D&cauthor=true&cauthor_uid=27185545)., [Guo, L](https://www.ncbi.nlm.nih.gov/pubmed/?term=Guo%20L%5BAuthor%5D&cauthor=true&cauthor_uid=27185545), [Zhu, L](https://www.ncbi.nlm.nih.gov/pubmed/?term=Zhu%20L%5BAuthor%5D&cauthor=true&cauthor_uid=27185545). et al. (2016). The rice thylakoid membranebound ascorbate peroxidase OsAPX8 functions in tolerance to bacterial blight*. [Sci Rep.](https://www.ncbi.nlm.nih.gov/pubmed/?term=The+rice+thylakoid+membrane-bound+ascorbate+peroxidase+OsAPX8+functions+in+tolerance+to+bacterial+blight)*  17(6), 26104. doi: 10.1038/srep26104.

# [Jisha, V](https://www.ncbi.nlm.nih.gov/pubmed/?term=Jisha%20V%5BAuthor%5D&cauthor=true&cauthor_uid=26035591)., [Dampanaboina, L](https://www.ncbi.nlm.nih.gov/pubmed/?term=Dampanaboina%20L%5BAuthor%5D&cauthor=true&cauthor_uid=26035591)., [Vadassery, J](https://www.ncbi.nlm.nih.gov/pubmed/?term=Vadassery%20J%5BAuthor%5D&cauthor=true&cauthor_uid=26035591)., [Mithofer, A](https://www.ncbi.nlm.nih.gov/pubmed/?term=Mith%C3%B6fer%20A%5BAuthor%5D&cauthor=true&cauthor_uid=26035591)., [Kappara, S](https://www.ncbi.nlm.nih.gov/pubmed/?term=Kappara%20S%5BAuthor%5D&cauthor=true&cauthor_uid=26035591)^.^, [Ramanan, R](https://www.ncbi.nlm.nih.gov/pubmed/?term=Ramanan%20R%5BAuthor%5D&cauthor=true&cauthor_uid=26035591). (2015) Overexpression of an AP2/ERF Type Transcription Factor OsEREBP1 Confers Biotic and Abiotic stress Tolerance in Rice. *[PLoS One](https://www.ncbi.nlm.nih.gov/pubmed/26035591)*. 10(6), e0127831. doi:10.1371/journal.pone.0127831.

# [Khong, G.N](https://www.ncbi.nlm.nih.gov/pubmed/?term=Khong%20GN%5BAuthor%5D&cauthor=true&cauthor_uid=26424158)., [Pati, P.K](https://www.ncbi.nlm.nih.gov/pubmed/?term=Pati%20PK%5BAuthor%5D&cauthor=true&cauthor_uid=26424158)., [Richaud, F](https://www.ncbi.nlm.nih.gov/pubmed/?term=Richaud%20F%5BAuthor%5D&cauthor=true&cauthor_uid=26424158)., [Parizot, B](https://www.ncbi.nlm.nih.gov/pubmed/?term=Parizot%20B%5BAuthor%5D&cauthor=true&cauthor_uid=26424158)., [Bidzinski, P](https://www.ncbi.nlm.nih.gov/pubmed/?term=Bidzinski%20P%5BAuthor%5D&cauthor=true&cauthor_uid=26424158)., [Mai, .C.D](https://www.ncbi.nlm.nih.gov/pubmed/?term=Mai%20CD%5BAuthor%5D&cauthor=true&cauthor_uid=26424158), et al., (2015). OsMADS26 Negatively Regulates Resistance to Pathogens and Drought Tolerance in Rice. *[Plant Physiol](https://www.ncbi.nlm.nih.gov/pubmed/?term=OsMADS26+Negatively+Regulates+Resistance+to+Pathogens+and+Drought+Tolerance+in+Rice1)*[.](https://www.ncbi.nlm.nih.gov/pubmed/?term=OsMADS26+Negatively+Regulates+Resistance+to+Pathogens+and+Drought+Tolerance+in+Rice1) 169, 2935-49. doi: 10.1104/pp.15.01192.

[Kuang, J](https://www.ncbi.nlm.nih.gov/pubmed/?term=Kuang%20J%5BAuthor%5D&cauthor=true&cauthor_uid=28900229)., [Liu, J](https://www.ncbi.nlm.nih.gov/pubmed/?term=Liu%20J%5BAuthor%5D&cauthor=true&cauthor_uid=28900229)., [Mei, J](https://www.ncbi.nlm.nih.gov/pubmed/?term=Mei%20J%5BAuthor%5D&cauthor=true&cauthor_uid=28900229)., [Wang, C](https://www.ncbi.nlm.nih.gov/pubmed/?term=Wang%20C%5BAuthor%5D&cauthor=true&cauthor_uid=28900229)., [Hu, H](https://www.ncbi.nlm.nih.gov/pubmed/?term=Hu%20H%5BAuthor%5D&cauthor=true&cauthor_uid=28900229)., [Zhang, Y](https://www.ncbi.nlm.nih.gov/pubmed/?term=Zhang%20Y%5BAuthor%5D&cauthor=true&cauthor_uid=28900229)., et al. (2017). A Class II small heat shock protein OsHsp18.0 plays positive roles in both biotic and abiotic defense responses in rice. *[Sci Rep.](https://www.ncbi.nlm.nih.gov/pubmed/?term=A+Class+II+small+heat+shock+protein+OsHsp18.0+plays+positive+roles+in+both+biotic+and+abiotic+defense+responses+in+rice)*7, 11333. doi: 10.1038/s41598-017-11882-x.

[Lee, H](https://www.ncbi.nlm.nih.gov/pubmed/?term=Lee%20H%5BAuthor%5D&cauthor=true&cauthor_uid=29330772)., [Cha, J](https://www.ncbi.nlm.nih.gov/pubmed/?term=Cha%20J%5BAuthor%5D&cauthor=true&cauthor_uid=29330772)., [Choi, C](https://www.ncbi.nlm.nih.gov/pubmed/?term=Choi%20C%5BAuthor%5D&cauthor=true&cauthor_uid=29330772)., [Choi, N](https://www.ncbi.nlm.nih.gov/pubmed/?term=Choi%20N%5BAuthor%5D&cauthor=true&cauthor_uid=29330772)., [Ji, H.S](https://www.ncbi.nlm.nih.gov/pubmed/?term=Ji%20HS%5BAuthor%5D&cauthor=true&cauthor_uid=29330772)., [Park, S.R](https://www.ncbi.nlm.nih.gov/pubmed/?term=Park%20SR%5BAuthor%5D&cauthor=true&cauthor_uid=29330772)., et al. (2018). Rice WRKY11 Plays a Role in Pathogen Defense and Drought Tolerance. *[Rice](https://www.ncbi.nlm.nih.gov/pubmed/29330772" \o "Rice (New York, N.Y.).)* [(N Y).](https://www.ncbi.nlm.nih.gov/pubmed/29330772" \o "Rice (New York, N.Y.).)  11, 5. doi: 10.1186/s12284-018-0199-0.

# Li, N., Kong, L., Zhou, W., Zhang, X., Wei, S., Ding, X. et al. (2013). Overexpression of Os2H16 enhances resistance to phytopathogens and tolerance to drought stress in rice. [*Plant Cell*](https://link.springer.com/journal/11240)*.* 115, 429–441. doi:[10.1007/s11240-013-0374-3](https://doi.org/10.1007/s11240-013-0374-3).

# [Liu, N.N.,](http://www.sciencedirect.com/science/article/pii/S1672630809600044#!) [Ling, J.,](http://www.sciencedirect.com/science/article/pii/S1672630809600044#!) [Zhang, W.](http://www.sciencedirect.com/science/article/pii/S1672630809600044#!)W., [Liu, L.L., Zhai, H.Q.](http://www.sciencedirect.com/science/article/pii/S1672630809600044#!) and [Wan J.M.](http://www.sciencedirect.com/science/article/pii/S1672630809600044#!) (2008) Role of LOX3 Gene in Alleviating Adverse Effects of Drought and Pathogens in Rice. *Rice Science*. 15, 276–282. [doi. 10.1016/S1672-6308(09)60004-4](https://doi.org/10.1016/S1672-6308(09)60004-4).

# [Ma, H](https://www.ncbi.nlm.nih.gov/pubmed/?term=Ma%20H%5BAuthor%5D&cauthor=true&cauthor_uid=28857351)., [Chen, J](https://www.ncbi.nlm.nih.gov/pubmed/?term=Chen%20J%5BAuthor%5D&cauthor=true&cauthor_uid=28857351)., [Zhang, Z](https://www.ncbi.nlm.nih.gov/pubmed/?term=Zhang%20Z%5BAuthor%5D&cauthor=true&cauthor_uid=28857351)., [Ma, L](https://www.ncbi.nlm.nih.gov/pubmed/?term=Ma%20L%5BAuthor%5D&cauthor=true&cauthor_uid=28857351)., [Yang, Z](https://www.ncbi.nlm.nih.gov/pubmed/?term=Yang%20Z%5BAuthor%5D&cauthor=true&cauthor_uid=28857351)., [Zhang, Q](https://www.ncbi.nlm.nih.gov/pubmed/?term=Zhang%20Q%5BAuthor%5D&cauthor=true&cauthor_uid=28857351)., et al. (2017). MAPK kinase 10.2 promotes disease resistance and drought tolerance by activating different MAPKs in rice. *[Plant J.](https://www.ncbi.nlm.nih.gov/pubmed/28857351)* 92, 557-570 doi: 10.1111/tpj.13674.

[Nakashima, K](https://www.ncbi.nlm.nih.gov/pubmed/?term=Nakashima%20K%5BAuthor%5D&cauthor=true&cauthor_uid=17587305)., [Tran, L.S](https://www.ncbi.nlm.nih.gov/pubmed/?term=Tran%20LS%5BAuthor%5D&cauthor=true&cauthor_uid=17587305)., [Van-Nguyen, D](https://www.ncbi.nlm.nih.gov/pubmed/?term=Van%20Nguyen%20D%5BAuthor%5D&cauthor=true&cauthor_uid=17587305)., [Fujita, M](https://www.ncbi.nlm.nih.gov/pubmed/?term=Fujita%20M%5BAuthor%5D&cauthor=true&cauthor_uid=17587305)., [Maruyama, K](https://www.ncbi.nlm.nih.gov/pubmed/?term=Maruyama%20K%5BAuthor%5D&cauthor=true&cauthor_uid=17587305)., [Todaka, D](https://www.ncbi.nlm.nih.gov/pubmed/?term=Todaka%20D%5BAuthor%5D&cauthor=true&cauthor_uid=17587305). et al. (2007). Functional analysis of a NAC-type transcription factor OsNAC6 involved in abiotic and biotic stress-responsive gene expression in rice. [*Plant J*.](https://www.ncbi.nlm.nih.gov/pubmed/17587305) 51, 617-30. doi:[10.1111/j.1365-313X.2007.03168.x](https://doi.org/10.1111/j.1365-313X.2007.03168.x).

[Wang](https://www.ncbi.nlm.nih.gov/pubmed/?term=Wang%20Z%5BAuthor%5D&cauthor=true&cauthor_uid=28419172), Z.,  [Han](https://www.ncbi.nlm.nih.gov/pubmed/?term=Han%20Q%5BAuthor%5D&cauthor=true&cauthor_uid=28419172), Q.,  [Zi](https://www.ncbi.nlm.nih.gov/pubmed/?term=Zi%20Q%5BAuthor%5D&cauthor=true&cauthor_uid=28419172), Q.,  [Lv](https://www.ncbi.nlm.nih.gov/pubmed/?term=Lv%20S%5BAuthor%5D&cauthor=true&cauthor_uid=28419172), S., [Qiu](https://www.ncbi.nlm.nih.gov/pubmed/?term=Qiu%20D%5BAuthor%5D&cauthor=true&cauthor_uid=28419172), D., and [Zeng](https://www.ncbi.nlm.nih.gov/pubmed/?term=Zeng%20H%5BAuthor%5D&cauthor=true&cauthor_uid=28419172) H. (2017). Enhanced disease resistance and drought tolerance in transgenic rice plants overexpressing protein elicitors from *Magnaporthe oryzae*. [*PLoS One*](https://www.ncbi.nlm.nih.gov/pmc/articles/PMC5395183/). 12, e0175734. doi: [10.1371/journal.pone.0175734](https://dx.doi.org/10.1371%2Fjournal.pone.0175734)

# [Xiao, J](https://www.ncbi.nlm.nih.gov/pubmed/?term=Xiao%20J%5BAuthor%5D&cauthor=true&cauthor_uid=24130197), [Cheng, H](https://www.ncbi.nlm.nih.gov/pubmed/?term=Cheng%20H%5BAuthor%5D&cauthor=true&cauthor_uid=24130197), [Li, X](https://www.ncbi.nlm.nih.gov/pubmed/?term=Li%20X%5BAuthor%5D&cauthor=true&cauthor_uid=24130197), [Xiao, J](https://www.ncbi.nlm.nih.gov/pubmed/?term=Xiao%20J%5BAuthor%5D&cauthor=true&cauthor_uid=24130197), [Xu, C](https://www.ncbi.nlm.nih.gov/pubmed/?term=Xu%20C%5BAuthor%5D&cauthor=true&cauthor_uid=24130197) and [Wang, S](https://www.ncbi.nlm.nih.gov/pubmed/?term=Wang%20S%5BAuthor%5D&cauthor=true&cauthor_uid=24130197). (2013). Rice WRKY13 regulates cross talk between abiotic and biotic stress signaling pathways by selective binding to different cis-elements. [*Plant Physiol.*](https://www.ncbi.nlm.nih.gov/pubmed/24130197) 163, 1868-82. doi: 10.1104/pp.113.226019.

Xiong, L.Z. and Yang, Y.N. (2003). Disease resistance and abiotic stress tolerance in rice are inversely modulated by an abscisic acid-inducible mitogen-activated protein kinase. Plant Cell. 15, 745–759. doi: [10.1105/tpc.008714](https://dx.doi.org/10.1105%2Ftpc.008714).
